# Dynamic changes of mitral valve annulus geometry at preprocedural CT: relationship with functional classes of regurgitation

**DOI:** 10.1186/s41747-021-00231-3

**Published:** 2021-08-13

**Authors:** Anna Palmisano, Valeria Nicoletti, Caterina Colantoni, Caterina Beatrice Monti, Luigi Pannone, Davide Vignale, Fatemeh Darvizeh, Eustachio Agricola, Simone Schaffino, Francesco De Cobelli, Antonio Esposito

**Affiliations:** 1 Experimental Imaging Center, IRCCS San Raffaele Scientific Institute, Milan, Italy; 2 School of Medicine, Vita-Salute San Raffaele University, Milan, Italy; 3 Department of Biomedical Sciences for Health, Università degli Studi di Milano, Via Mangiagalli 31, 20133 Milan, Italy; 4 Echocardiography Unit, School of Medicine, IRCCS San Raffaele Scientific Institute, Milan, Italy; 5 Department of Radiology, IRCCS Policlinico San Donato, San Donato Milanese, Milan, Italy

**Keywords:** Computed tomography angiography, Heart valve prosthesis, Mitral valve, Mitral valve insufficiency, Planning techniques

## Abstract

**Background:**

We investigated mitral valve annular geometry changes during the cardiac cycle in patients with severe mitral regurgitation (MR) who underwent cardiac computed tomography angiography (CCTA) prior to percutaneous mitral valve replacement or annuloplasty.

**Methods:**

Fifty-one patients with severe MR and high surgical risk (Carpentier classification: 3 type I, 16 type II, 16 type IIIa, 16 type IIIb) underwent multiphase electrocardiographically gated (0–90%) CCTA, using a second generation dual-source CT scanner, as pre-procedural planning. Twenty-one patients without MR served as controls. The mitral valve annulus was segmented every 10% step of the R-R interval, according to the D-shaped segmentation model, and differences among groups were analysed by *t*-test or ANOVA.

**Results:**

Mitral annular area and diameters were larger in MR patients compared to controls, particularly in type II. Mitral annular area varied in MR patients throughout the cardiac cycle (mean ± standard deviation of maximum and minimum area 15.6 ± 3.9 cm^2^ *versus* 13.0 ± 3.5 cm^2^, respectively; *p* = 0.001), with greater difference between annular areas *versus* controls (2.59 ± 1.61 cm^2^ and 1.98 ± 0.6 cm^2^, *p* < 0.001). The largest dimension was found in systolic phases (20–40%) in most of MR patients (*n* = 27, 53%), independent of Carpentier type (I: *n* = 1, 33%; II: *n* = 10, 63%; IIIa: *n* = 8, 50%; IIIb: *n* = 8, 50%), and in protodiastolic phases (*n* = 14, 67%) for the control group.

**Conclusions:**

In severe MR, mitral annular area varied significantly throughout the cardiac cycle, with a tendency towards larger dimensions in systole.

## Key points

- At multiphase cardiac computed tomography angiography (CCTA), mitral valve annulus area showed greater modifications throughout the cardiac cycle in patients with mitral regurgitation than in the control group.
- Mitral valve prolapse was characterised by larger annulus dimensions and greater changes in mitral valve area over the cardiac cycle at CCTA in comparison to the other functional regurgitation types.
- The maximum annular area was mainly recorded within 20–40% of the cardiac cycle at CCTA.

## Background

Mitral valve regurgitation (MR) is the second most frequent valvular dysfunction in Europe, after aortic stenosis [1, 2]. It is estimated that around 10% of population aged over 75 have moderate to severe MR [3]. It can be caused by structural abnormality of one or more components of the mitral valve (primary MR), or by the distortion of the mitral valve apparatus caused by left ventricle (LV) and/or left atrium (LA) remodelling (secondary MR) [4]. Severe MR is a major source of morbidity and mortality worldwide and a frequent cause of heart failure, being associated with unfavourable prognosis when remains untreated [5]. Despite surgery is the treatment of choice for patients with severe MR, approximately 50% of patients are not eligible for surgery due to comorbidities [3, 6]. In the recent years, transcatheter mitral valve implantation (TMVI) is emerging as an effective alternative to surgery in high-risk surgical candidates [3]. TMVI revolutionised the management of mitral valve repair/replacement percutaneously based on MR aetiology and patients’ anatomical and clinical characteristics [7, 8]. Among the available treatment options, mitral valve replacement and annuloplasty have been considered in case of unfavourable anatomy for transcatheter mitral valve edge-to-edge repair. In fact, the preprocedural verification of anatomical suitability is crucial for the feasibility of the procedure. Correct mitral annulus sizing has been shown to be a major determinant for the procedure outcome to avoid severe complications due to oversizing (*e.g.*, left ventricle outflow tract obstruction, annular rupture) or undersizing (*e.g.*, prosthesis instability, paravalvular leak, residual regurgitation) [9, 10]. Echocardiography is the first line investigation to assess MR severity and its aetiology and for preprocedural planning and intraprocedural guidance [8]. In particular, transthoracic echocardiography (TTE) provides a quantitative assessment of MR severity, determines the different MR mechanism, and evaluates ventricular volume and function [11]. However, it is operator-dependent and can be affected by limited spatial resolution; it may also result inconclusive in patients with inadequate acoustic window. Preprocedural two-dimensional and three-dimensional transesophageal echocardiography (TEE) are essential to assess anatomical detail of mitral valve remodelling [11]. It can also be utilised in patients with inconclusive TTE or in case of inadequate acoustic window for a more accurate estimation of MR severity and mechanism [11].

Cardiac computed tomography angiography (CCTA) plays a complementary role to echocardiography for TMVI preprocedural planning [12], providing additional and more precise anatomical data [11]. CCTA provides isotropic volumes with high spatial and temporal resolution allowing highly detailed anatomical information of mitral valve and of cardiac structures over the entire cardiac cycle, providing information about their dynamic changes [11, 12]. Therefore, CCTA is of pivotal importance for accurate and reliable device sizing, for characterisation of the landing zone and the surrounding structures (*e.g.*, calcification, left ventricle outflow tract, coronary arteries, coronary sinus), and furthermore for the assessment of vascular access [12–15]. Based on these facts, CCTA is becoming increasingly important in TMVI planning [11, 14, 16, 17].

Considering the importance of correct annular sizing for final outcome, there are several studies investigating the three-dimensional mitral apparatus geometry and its variations during the cardiac cycle in healthy individuals and in pathological condition using echocardiography [18–22], with discordant results mainly due to limited sample size, heterogeneous methods (bidimensional, three-dimensional TTE, TEE), and patients’ features. Despite CCTA is more accurate and highly reproducible than echocardiography, only few studies are available on mitral valve geometry changes which are mainly focused on mitral valve prolapse [23–28]. These studies reported discordant results regarding the cardiac phases with larger annular dimension (systole *versus* diastole) and the dynamic changes throughout the cardiac cycle (increased *versus* reduced dynamic changes). A comparative CT study of mitral valve geometry changes over the cardiac cycle in patients with severe MR and in patients without MR may clarify the pathological alteration of valvular geometry and dynamics occurring in relation to different functional MR mechanism.

The aim of the present study is to evaluate the changes of mitral valve annular geometry throughout cardiac cycle in patients with severe MR with different aetiologies, who underwent pre-procedural CCTA prior to percutaneous mitral valve replacement or annuloplasty.

## Methods

### Study population

This retrospective single-centre study was approved by the Institutional Review Board, and informed consent was signed. From January 2017 to October 2020, seventy-two patients with severe symptomatic MR (age > 18 years), with high surgical risk, were candidates for percutaneous mitral valve replacement or annuloplasty according to the 2017 ESC/EACTS guidelines [29]. They underwent a preprocedural ECG-gated cardiac CT with retrospective multiphase acquisition (0–90% of the cardiac cycle). Patients with previous mitral valve surgery (*n* = 12), previous aortic surgery (*n* = 6), and degraded image quality due to respiratory motion artefacts (*n* = 3) were excluded.

**Fig. 1:**
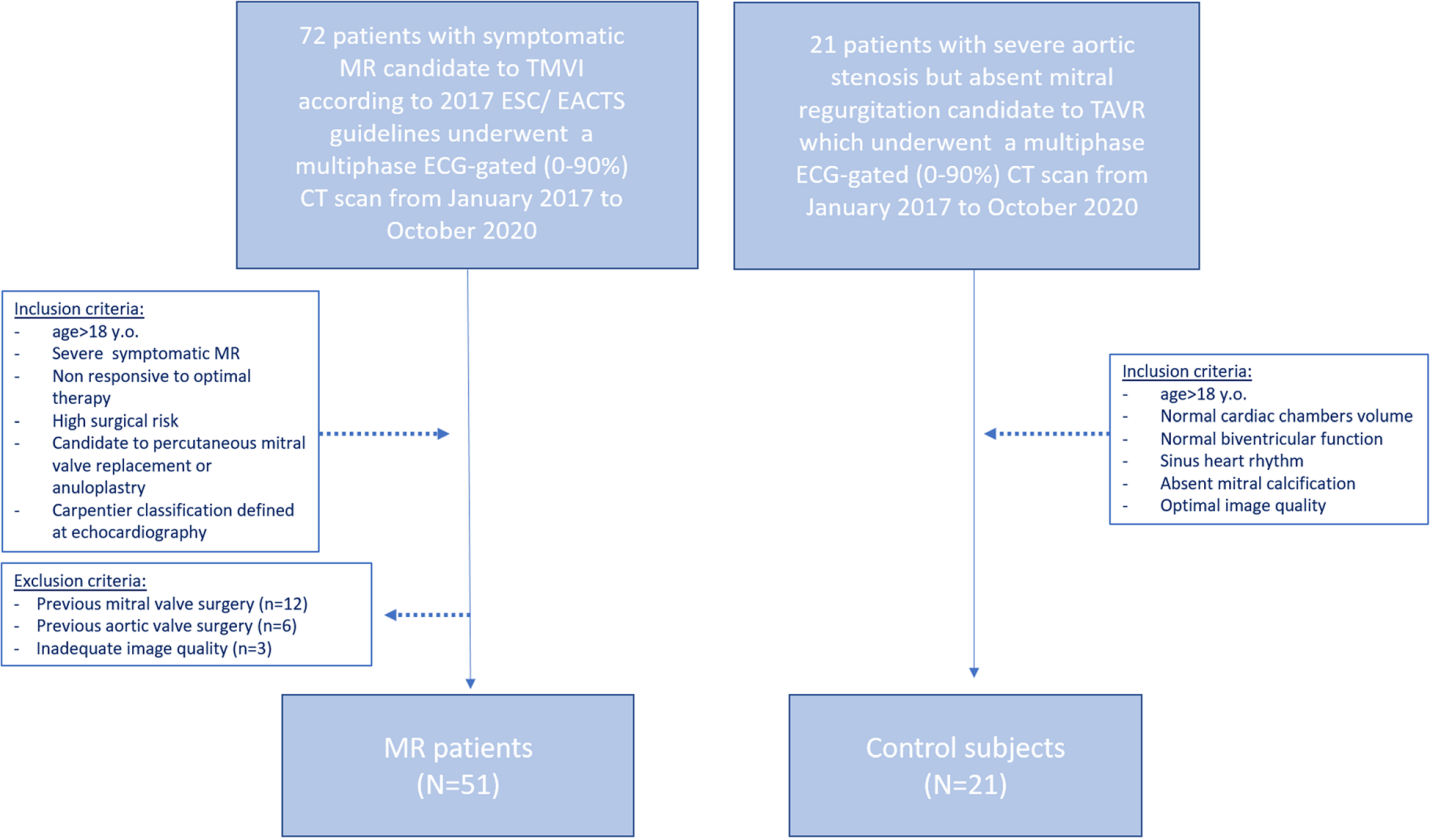
Enrolment flowchart

Twenty-one patients with severe aortic stenosis, without MR and any previous valve or cardiac surgery who underwent retrospective multiphase ECG-gated cardiac CT (0–90% phases of the cardiac cycle) for TAVR planning, were considered as the control group. Additional inclusion criteria for the control group were preserved EF, normal sinus rhythm, absent mitral valve calcification and/or the aorto-mitral curtain, and optimal image quality (without breathing artefact). Enrolment flowchart is reported in Fig. 1.

### CCTA acquisition protocol

All cardiac scans were performed using a II generation dual-source scanner (Somatom Definition Flash, Siemens Healthineers, Erlangen, Germany) with a retrospective ECG-gated acquisition, using the automatic tube voltage and current selection systems (CARE kV and CARE Dose4D, Siemens Healthineers), with reference tube voltage of 120 kVp and reference tube current-time product of 350 mAs/rotation. Gantry rotation time was 0.28 s, and helical pitch was automatically selected according to patients’ heart rate. All cardiac CT scan were synchronised with the first pass of contrast agent using automatic bolus-tracking method. Iodinated contrast media (Visipaque 320, General Electric Healthcare, Chicago, IL, USA) was administered using triphasic injection scheme: 70 mL contrast agent, followed by 40 mL of mixed solution (25% contrast agent and 75% saline), followed by further 40 mL of pure saline (total volume 80 mL).

Patients with severe MR and depressed ejection fraction showed limited tolerance to negative chronotropic/inotropic agent. Therefore, low-dose beta-blocker or diltiazem could be considered only in patients with heart rate above 100 bpm [30]. Since heart rate was under 100 bpm in all patients (72 ± 13 bpm in MR group and 68 ± 9 bpm in control group), no premedication for heart rate control was administered prior to CCTA acquisition [30].

Multiphase images were reconstructed in standard fashion at every 10% of the R-R interval (0–90%), at a slice thickness of 0.6 mm with an increment of 0.5 mm, using smooth kernel (I36) and an iterative reconstruction algorithm (SAFIRE, strength 2, Siemens Healthineers).

### CCTA image analysis

Image analysis was performed independently by two radiologists with different level of experience in cardiovascular imaging (D.V. and A.P. with 3 and 10 years of experience, respectively) blinded to clinical data, using reformatted reconstructions of the annulus plane using a dedicated software (Philips Intellispace Portal v8.0, Amsterdam, The Netherlands).

**Fig. 2:**
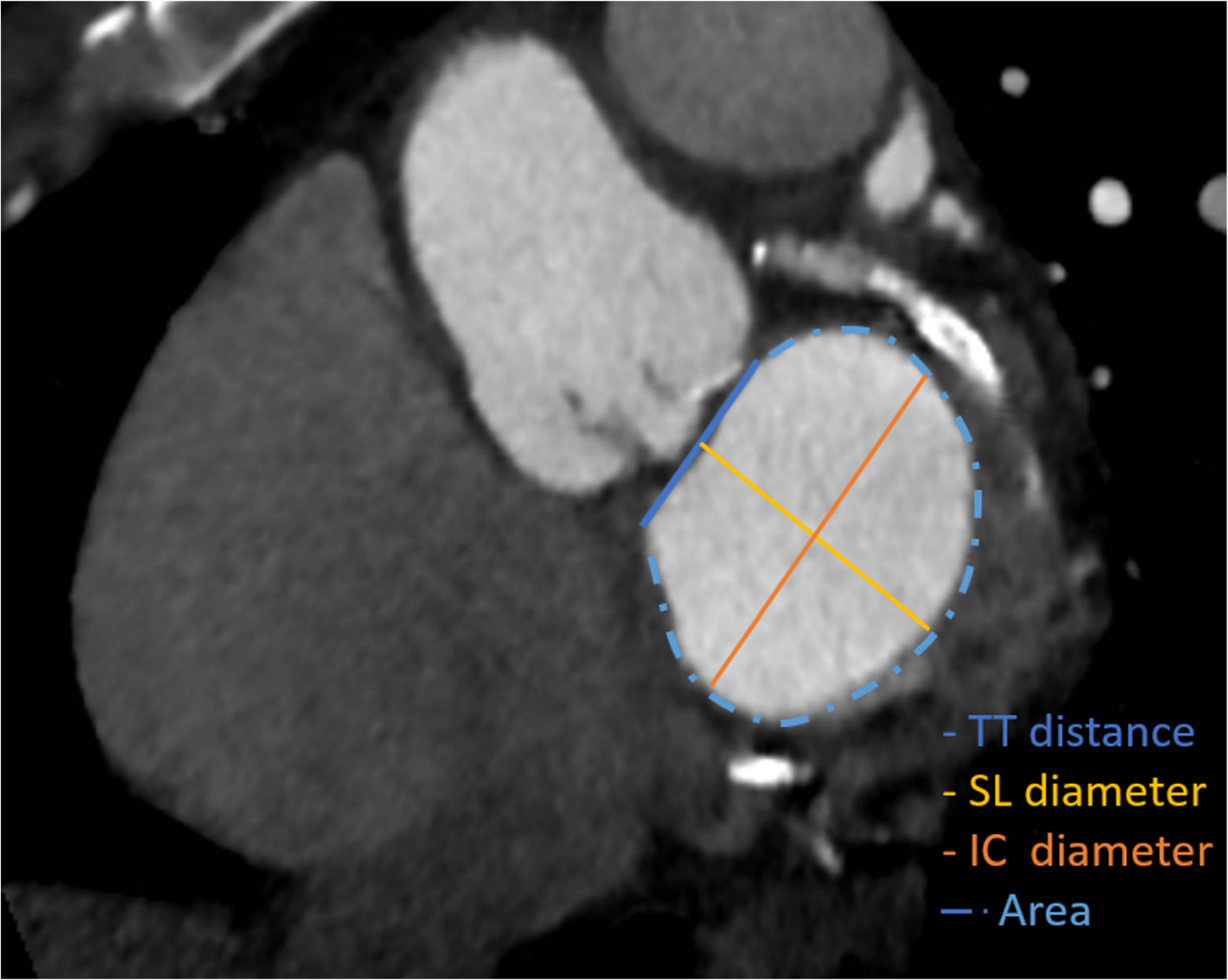
D-shaped segmentation of mitral annulus on cardiac computed tomography angiography (CCTA). Traced in blue is the trigone-to-trigone diameter, in yellow the septal-to-lateral diameter, in orange the intercommissural diameter, and the dashed light blue line plus the trigone-to-trigone diameter depict the mitral annulus

According to the D-shaped segmentation model (Fig. 2), the mitral valve annulus area and diameters [trigone-to-trigone distance (TT), intercommissural diameter (IC), and septal-to-lateral diameter (SL)] were measured every 10% of the entire R-R interval [23, 31, 32], in order to assess their dynamic changes. Furthermore, automatic segmentation of cardiac chamber has been performed with the calculation of left ventricular ejection fraction (LV-EF), left ventricular end-diastolic volume (LV-EDV), and left atrial end-systolic volume (LA-ESV).

### Echocardiography

Preprocedural transesophageal echocardiographic (TOE) study was performed in all patients. Images were obtained with GE Vivid E9 or GE Vivid E95 (General Electric Healthcare, Chicago, IL) and post-processed with the EchoPAC® General Electric software by two physicians (L.P. and E.A.) in consensus. LV-EF, LV-EDV, and LA-ESV were measured following the ASE guidelines [33].

**Fig. 3:**
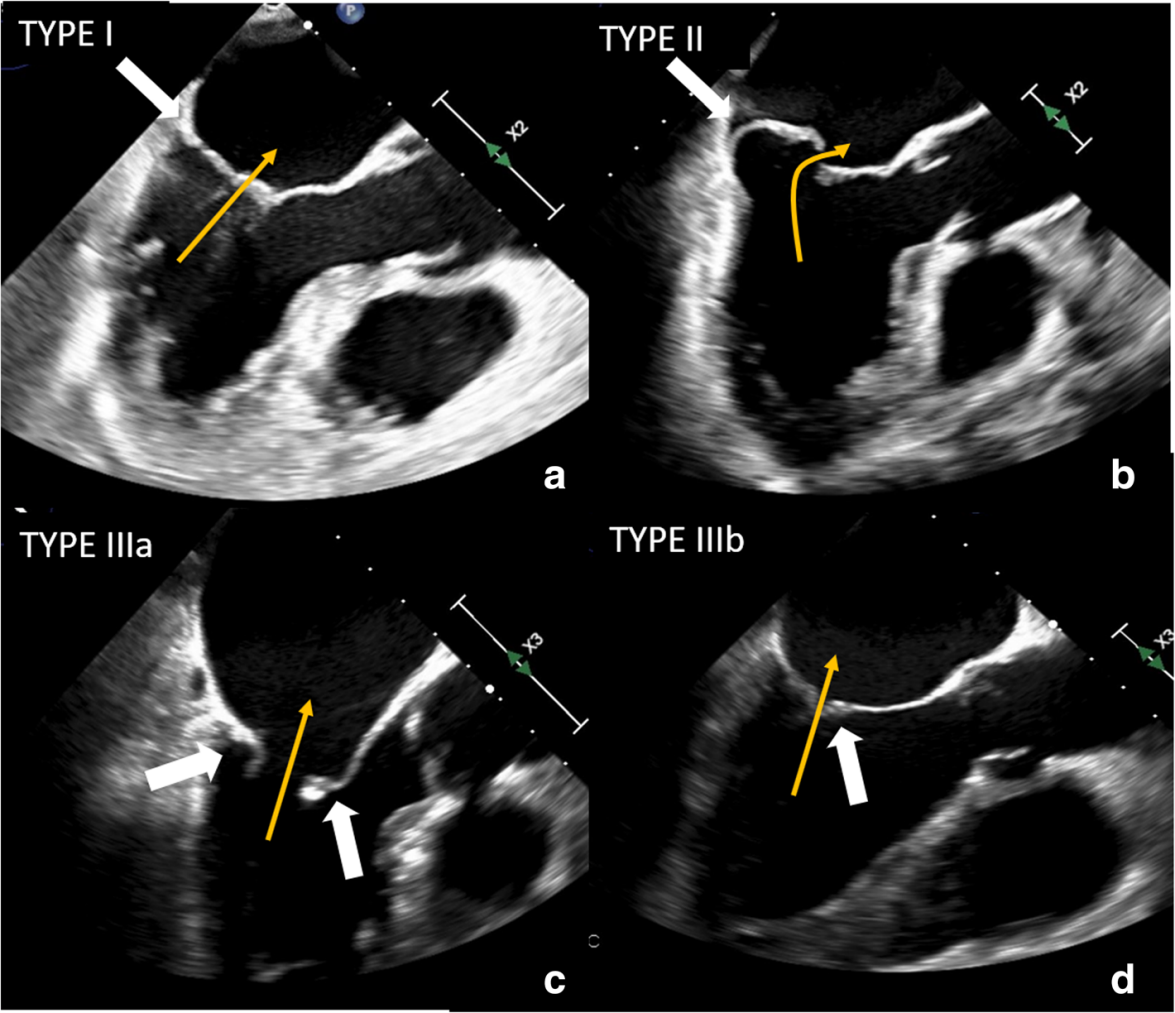
Transesophageal echocardiographic (TOE) images of mitral regurgitation (MR) according to the Carpentier classification. The MR flow direction is represented by an orange arrow. (**a**) An example of Carpentier type I: long-axis midesophageal (ME) view TOE showing normal valve anatomy with no prolapse or tethering but atrial dilatation/dysfunction (atrial MR) with horizontal coaptation of the leaflet (white arrow). (**b**) An example of Carpentier type II: prolapse of posterior leaflet (white arrow) in a valve with myxomatous degeneration (Barlow’s disease). (**c**) Carpentier type IIIa. Long-axis ME view TOE showing diastolic movement restriction (white arrows) in a valve affected by rheumatic disease. Of note, leaflet thickening and retraction with “hockey stick” morphology of anterior leaflet (white arrow on the right side). (**d**) Carpentier type IIIb. Long-axis ME view TOE showing systolic movement restriction in a patient with previous anterior myocardial infarction. Of note, the coaptation gap between mitral valve leaflets (white arrow)

Carpentier classification of mitral valve regurgitation based on leaflet motion was performed [34–36], and patients were classified as type I (normal leaflet motion and position), type II (excessive leaflet motion), type IIIa (restricted leaflet motion in both systole and diastole), and type IIIb (restricted leaflet motion only in systole) (Fig. 3). In detail, type I includes patients with leaflet perforation or cleft and patients with atrial fibrillation or with non-ischemic cardiomyopathy; type II includes patients with mitral valve prolapse, due to Barlow’s disease or fibroelastic deficiency, or patients with cordal or papillary muscle elongation or rupture; type IIIa includes patients with leaflet thickening and/or retraction, cordal thickening and/or shortening, and commissural fusion like in cases of rheumatic mitral valve disease, mitral annular calcification, and drug-induced mitral valve disease; type IIIb includes patients with impairment of LV function with annulus dilatation and papillary muscle displacement for example in case of ischemic cardiomyopathy.

### Statistical analysis

Continuous variables were expressed as mean ± standard deviation and categorical variables as percentage. Changes in the mitral annular dimensions throughout each phase of the cardiac cycle (0–90%) were analysed in each patient’s group and expressed as mean ± standard deviation.

Maximum and minimum mitral annulus area and annular dimensions between MR and control groups were compared using Student’s *t* test (normality was assessed by Kolmogorov-Smirnov test).

Differences in mitral annulus area and diameters among Carpentier’s classes and control group were evaluated using analysis of variance (ANOVA). Bonferroni tests were used as a post hoc analysis among the groups. Correlation analysis between mitral annulus area and clinical parameters (age, gender, BSA), and cardiac chamber volumes and function on CCTA was assessed using Pearson or Spearman correlation tests. To evaluate the agreement and reproducibility of the measurement between the two readers, the intraclass correlation coefficient (ICC) was used (ICC excellent 0.75–1.00; good 0.60–0.74; fair 0.40–0.59; and poor < 0.40) [37]. Statistical analyses were performed using SPSS Statistics Version 20 (IBM, Armonk, NY); *p*-values < 0.05 were considered statistically significant.

## Results

### Patients’ characteristics

Baseline characteristics of patients with MR and control subjects are reported in Table 1.

**Table 1:** Baseline patients’ characteristics

	MR patients (total = 51)	Control subjects (total = 21)
**Patient characteristics**		
Age, years	72 ± 14	80 ± 6
Males, *n* (%)	27 (53%)	9 (43%)
Body mass index, kg/m^2^	24.5 ± 5.2	26 ± 6
Body surface area, m^2^	1.77 ± 0.24	1.73 ± 0.25
Atrial fibrillation, *n* (%)	30 (59%)	0 (0%)
**Carpentier classification**		
Type I, *n* (%)	3 (5.9%)	-
Type II, *n* (%)	16 (31.4%)	-
Type IIIa, *n* (%)	16 (31.4%)	-
Type IIIb, *n* (%)	16 (31.4%)	-

Continuous variables are reported as mean ± standard deviation, except otherwise specified

*MR* Mitral valve regurgitation

Patients with MR were mainly men (*n* = 27, 53%) with a mean age of 72 ± 14 years old. They were classified as follows: 3 patients (5.8%) with Carpentier type I, 16 patients (31.4%) with type II, 16 patients (31.4%) with type IIIa, and 16 patients (31.4%) with type IIIb. Most of the patients with MR (*n*=30, 59%) had atrial fibrillation. Control group included 21 subjects with mean age of 80 ± 6 years old, and among them, 43% (9/21) were male.

### Mitral annulus geometry

**Fig. 4:**
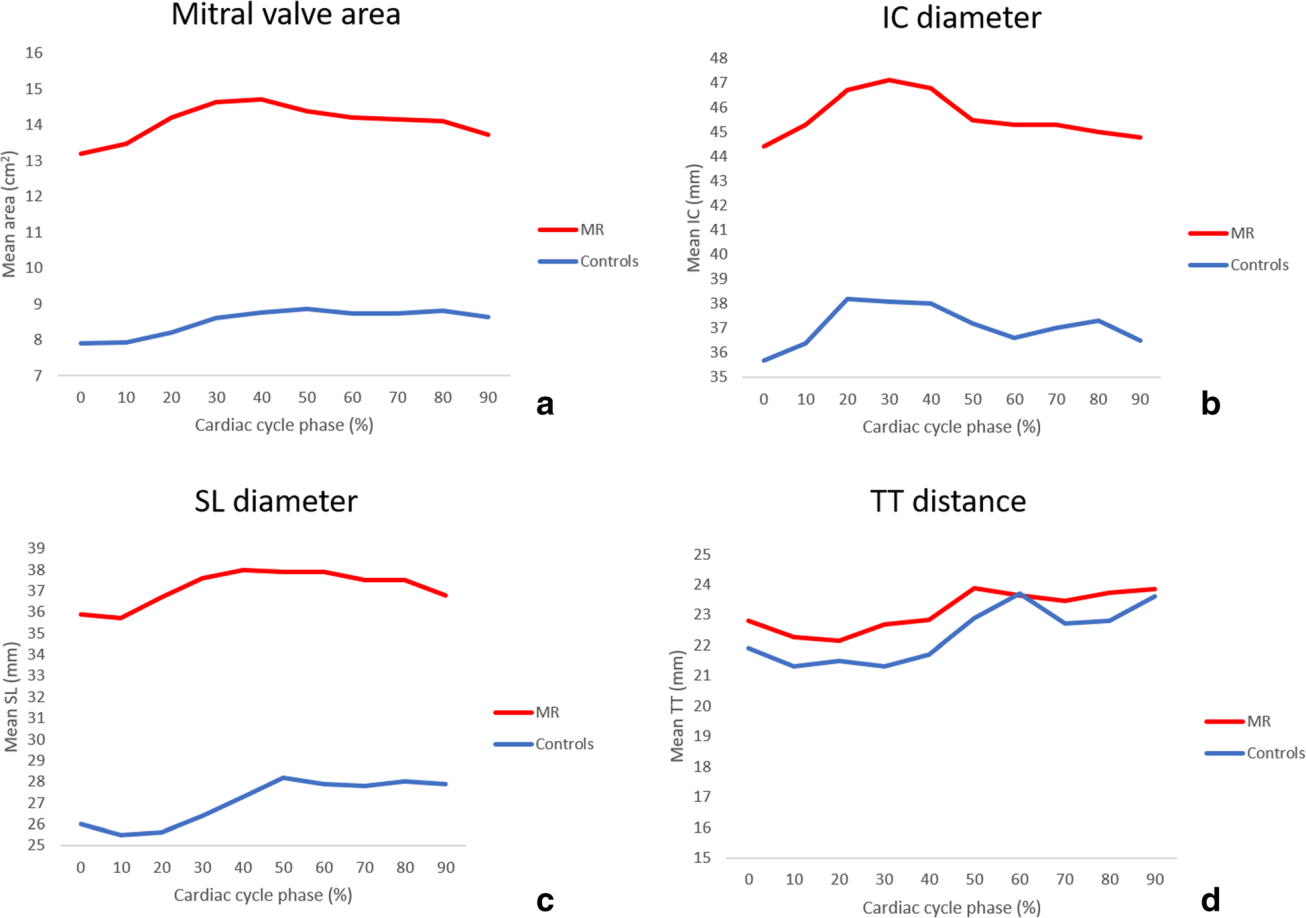
Mitral valve dimension modification during the cardiac cycle in mitral regurgitation and control patients

Table 2 and Fig. 4 show the minimum and maximum annulus area, IC, SL, and TT diameters in MR and control groups as well as its changes throughout the cardiac cycle. Maximum area and diameters were significantly larger in MR patients in comparison to control subjects (*p* < 0.001) except for TT distance (*p* = 0.207, Table 2). The SL/IC ratio, index of ellipticity, was greater in MR than in control patients (0.82 ± 0.09 *versus* 0.73 ± 0.05; *p* < 0.001).

**Table 2:** Left cardiac chamber volumes and mitral annulus metrics in MR and control subjects

	MR group (total = 51)	Control group (total = 21)	***p*** value
**Volumes and function at Echo**			
Left atrium ESV, mL	131 ± 55	62 ± 12	< 0.001
Left ventricle EDV, mL	161 ± 81	83 ± 23	< 0.001
Ejection fraction, %	44.4 ± 15.7	63.6 ± 5.8	< 0.001
**Volumes and function at CCTA**			
Left atrium ESV, mL	202.7 ± 71.3	107.7 ± 24.3	< 0.001
Left ventricle EDV, mL	240.5 ± 110.3	135.6 ± 29.1	< 0.001
Ejection fraction, %	40.7 ± 15.0	68.7 ± 7.4	< 0.001
**Mitral valve geometry at CCTA**			
D-shaped area max, cm^2^	15.6 ± 3.9	9.5 ± 1.8	< 0.001
D-shaped area min, cm^2^	13.0 ± 3.5	7.6 ± 1.6	< 0.001
TT distance max, mm	26.5 ± 4.1	25.3 ± 3.1	0.207
IC diameter max, mm	48.6 ± 6.4	39.6 ± 3.1	< 0.001
SL diameter max, mm	40.0 ± 5.5	29.7 ± 3.4	< 0.001
SL/IC ratio	0.82 ± 0.09	0.73 ± 0.05	< 0.001
Valve calcium volume, cm^3^	729.7 ± 1,994.1	0	< 0.001

Continuous variables are reported as mean ± standard deviation

*CCTA* cardiac CT angiography, *EDV* end diastolic volume, *MR* mitral valve regurgitation, *TT* trigone-to-trigone distance, *IC* intercommissural diameter, *SL* septal-to-lateral diameter

MR Carpentier types showed significantly different annular area (*p* = 0.002), IC (*p* = 0.002), and SL (*p* = 0.013) diameters (Table 3). Carpentier type II showed largest area (18.0 ± 4.5 cm^2^), IC diameter (52.6 ± 6.4 mm), and SL diameter (42.5 ± 6.4 mm), however, with only significant difference compared with Carpentier type IIIa (*p* = 0.001 for each parameters).

**Table 3:** Mitral annular dimensions in the different types of MR based on Carpentier’s classification

	Type I (total, 3)	Type II (total, 16)	Type IIIa (total, 16)	Type IIIb (total, 16)	***p*** value
**Mitral valve geometry**					
D-shaped area max, cm^2^	15.9 ± 7.8	18.0 ± 4.5^a^	12.9 ± 2.4^a^	15.8 ± 3.2	0.002
Maximum TT distance, mm	30.3 ± 7.9	27.5 ± 4.5	25.4 ± 3.5	26.0 ± 2.9	0.152
Maximum IC diameter, mm	47.9 ± 3.1	52.6 ± 6.4^a^	44.4 ± 5.0^a^	49.0 ± 5.6	0.002
Maximum SL diameter, mm	41.4 ± 2.3	42.5 ± 6.0^a^	36.5 ± 5.0^a^	40.8 ± 4.4	0.013
Calcification volume, cm^3^	0 ± 0	635 ± 2,031	1,331± 2,551	360 ± 1,106	0.469
Maximum area in 20–40% cycle	1/3, 33%	10/16, 63%	8/16, 50%	8/16, 50%	-
Maximum area in 60–80% cycle	1/3, 33%	3/16, 19%	5/16, 31%	3/16, 19%	-

Data are reported as mean ± standard deviation

*MR* Mitral valve regurgitation, *TT* Trigone-to-trigone distance, *IC* Intercommissural diameter, *SL* Septal-to-lateral diameter

^a^At post hoc analysis significant differences (*p* = 0.001 for each parameter)

No differences were reported in TT diameter among each Carpentier types (*p* = 0.152).

The maximum annulus area in MR group was correlated to male sex (*r* = 0.402, *p* = 0.003), LV-EDV (*r* = 0.378, *p* = 0.006), and LA-ESV (*r* = 0.565, *p* < 0.001). It was not significantly correlated with age (*r* = −0.224, *p* = 0.114), BSA (*r* = 0.052, *p* = 0.765), LV-EF (*r* = 0.089, *p* = 0.536), and atrial fibrillation (*r* = 0.062, *p* = 0.670). In the control group, the annulus area was significantly correlated with BSA (*r* = 0.71, *p* = 0.001), male sex (*r* = 0.60, *p* = 0.005), LV-EDV (*r* = 0.73, *p* = 0.001), and LA-ESV (*r* = 0.52, *p* = 0.020) but not with LV-EF (*r* = 0.14, *p* = 0.547).

### Mitral annulus dynamic changes in the cardiac cycle (0–90%)

**Fig. 5:**
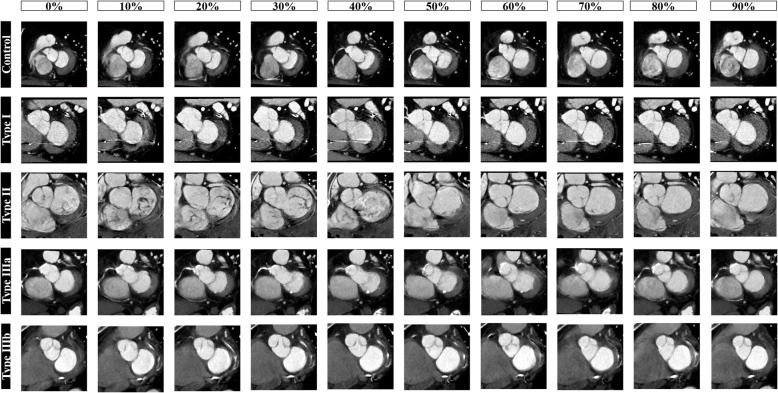
Short-axis reformatted cardiac computed tomography angiography images of the mitral valve annulus in each cardiac phase in exemplifying control patient and in mitral regurgitation patients according to Carpentier classification

**Fig. 6:**
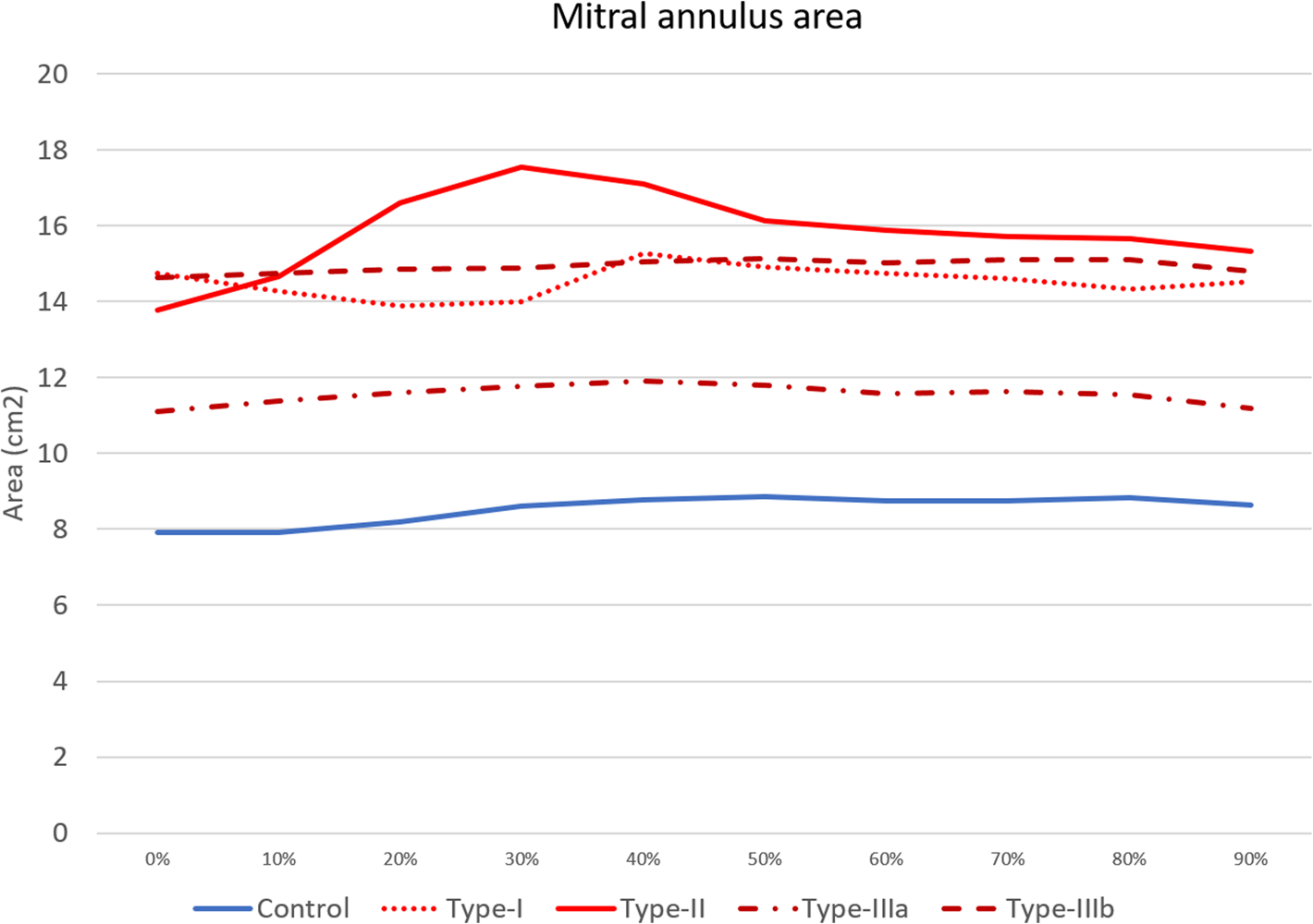
Dynamic modifications of the mitral annulus area through the different phases of the cardiac cycle according to Carpentier classification of mitral regurgitation and in control patients

Exemplifying cases of mitral valve annulus modification in the cardiac cycle in control subjects and Carpentier MR classes are reported in Fig. 5. The maximum and minimum value of the mitral annulus area across the entire cardiac cycle (0–90%) were significantly different in the MR group (15.6 ± 3.9 *versus* 13.0 ± 3.5 cm^2^ respectively, *p* = 0.001) as well as in the control group (9.62 ± 1.76 cm^2^ *versus* 7.60 ± 1.65 cm^2^ respectively, *p* < 0.001), with greater difference in MR patients compared to control subject (2.59 ± 1.61 *versus* 1.98 ± 0.6 cm^2^, *p* < 0.001). This result is driven mainly by Carpentier type II patients, which showed greater difference between maximum and minimum annulus areas (3.73 ± 2.25 cm^2^; *p* < 0.001) (Table 4 and Fig. 6).

**Table 4:** Difference between maximal and minimal annular area measured through the cardiac cycle in control subjects and MR patients based on Carpentier’s classification

	Control group	MR total	MR type I	MR type II	MR type IIIa	MR type IIIb	***p*** value
**Δ Area** **max-min, cm**^2^	1.98 ± 0.6	2.59 ± 1.61	2.26 ± 0.1	3.73 ± 2.25	2.39 ± 1.0	1.7 ± 0.52	< 0.001

Data are reported as mean ± standard deviation

*MR* Mitral valve regurgitation

**Fig. 7:**
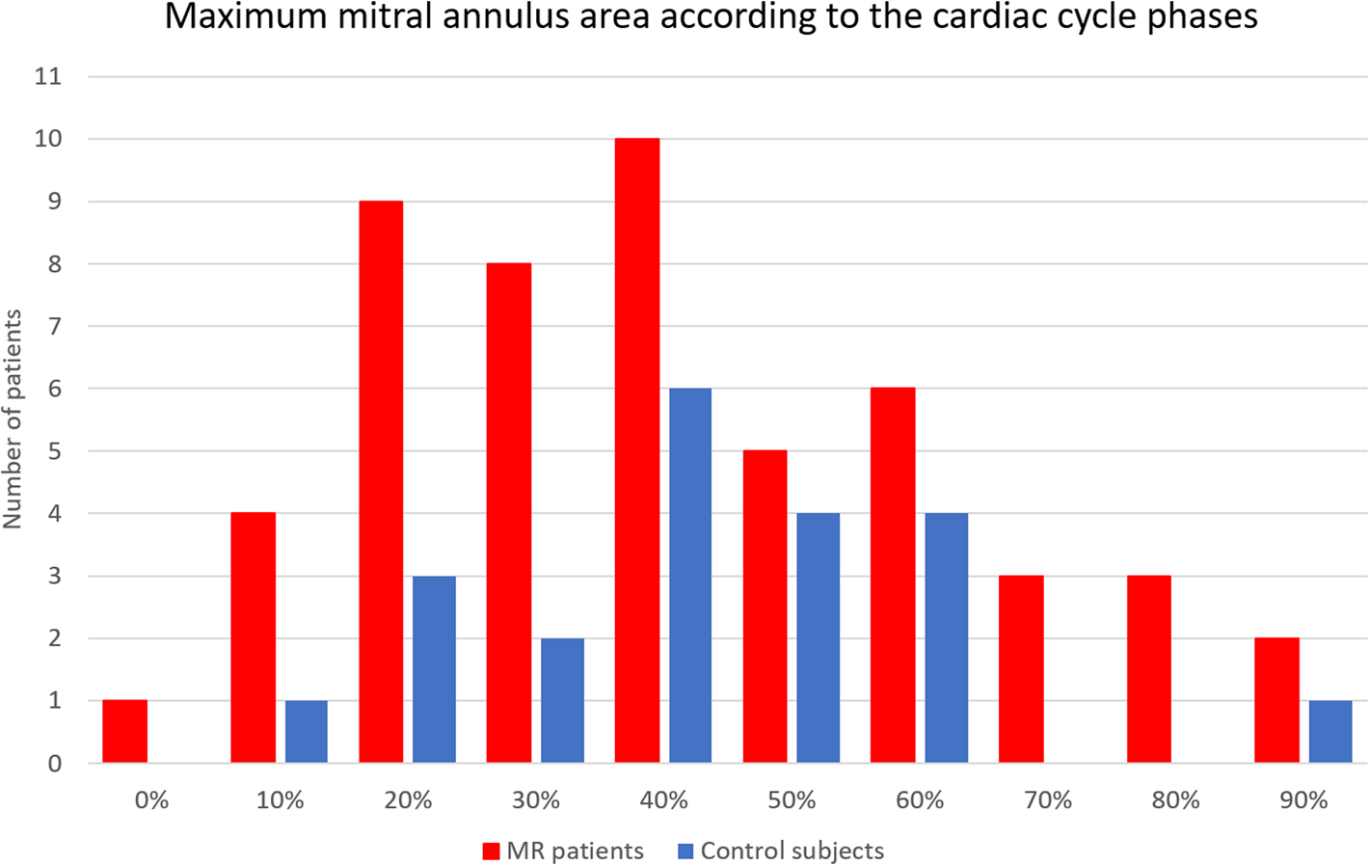
Distribution of maximum mitral annular area in mitral regurgitation and control patients

Less dynamic changes were observed in types I, IIIa, and IIIb across the cardiac cycle (Fig. 6). In the MR group, the minimum valvular area was found mainly at 0–20% of cardiac cycle (23/51 patients, 45%) while the maximum area was mainly observed at 20–40% phases of cardiac cycle (*n* = 27, 53%) (Fig. 7). This was due to larger annulus area in systolic phase (20–40%) in type II, type IIIa, and type IIIb, while type I MR showed random distribution of the maximum area.

In the control group, the minimum and maximum areas were more homogeneously distributed, with minimum area mostly found in 0–20% phases of the cardiac cycle (*n* = 14, 67%) and the maximum area mainly in protodiastolic phases (40–60% of the cardiac cycle, *n* = 14, 67%) (Fig. 7).

### Interobserver agreement

The interobserver agreement was excellent for all the analysed parameters: annular area (ICC 0.94, 95% confidence interval (CI), 0.91–0.95), IC diameter (ICC 0.91, 95% CI, 0.89–0.93), SL diameter (ICC 0.91, 95% CI 0.86–0.94), and TT distance (ICC 0.87, 95% CI 0.78–0.96).

## Discussion

Correct mitral annulus measurement is of pivotal importance for device sizing and for a successful TMVI procedure [10, 15]. The understanding of the dynamic changes of the mitral annulus geometry across the cardiac cycle has implications on landing zone and device delivery [38].

Previous studies showed discordant results regarding mitral valve geometry changes in patients with MR [14, 16, 17, 28]. Therefore, an improvement in understanding of mitral valve geometry and dynamic changes across the cardiac cycle in relation to the different functional aetiologies of MR is of great importance to improve CCTA scanning protocol in order to improve image analysis and reduce radiation exposure.

To the best of our knowledge, this is the first study providing a multiphase CCTA characterisation of mitral annulus geometry in patients with severe MR of different functional aetiologies.

Main findings of the present study were mitral valve annulus was significantly enlarged in severe MR patients (*p* < 0.001), especially in mitral valve prolapse (Carpentier type II), which were also characterised by greater changes of mitral annulus area and diameters during the cardiac cycle (*p* = 0.002, *p* = 0.002, and *p* = 0.013 for mitral annulus area, IC, and SL diameters, respectively). Largest annular area was mainly observed in systolic phases (20–40% phases) in severe MR patients (53%), differently from control subjects with largest annular area mostly found in protodiastolic phases (40–60% phases, 67% of patients). Finally, the mitral annulus area changed significantly during the cardiac cycle, independently from the presence of MR, with the greatest change in Carpentier type II.

Our findings are in accordance with previous studies on echocardiography [20, 22, 39, 40] and CCTA [23–25] that demonstrated a significant mitral valve annulus enlargement in patients with mitral valve prolapse (Carpentier type II) and moderate-to-severe MR. This is probably related to both an increased outward tension during systole caused by excessive valve tissue and to mitral annular disjunction that causes the displacement of the posterior leaflet and annulus into the left atrium.

Some studies suggested a correlation between mitral valve area and left cardiac chamber volumes in the end-stage MR, independent of different leaflet motion alterations [20, 23, 25, 39, 40]; our results confirmed the aforementioned findings. Similarly to the control group, in MR patients, larger annular area was found in men [23, 25, 41, 42], while no significant correlation was achieved with BSA, which is likely due to the massive annular dilation and end-stage mitral valve apparatus remodelling in the patients with severe MR.

Larger mitral annulus area was found mostly in systolic phases in type II and types IIIa and IIIb MR patients, differently from control subjects in which larger dimension was mainly observed in early- to mid-diastolic phases (40–60%, *n*=14, 67% of cases). This finding is in accordance with a previous CCTA study [24]. Larger systolic dimensions in MR patients is likely due to systolic annular overexpansion and/or disjunction in type II [18, 25, 43] and annulus enlargement with systolic contractility dysfunction in type III MR patients [35]. Patients with mitral prolapse showed greater changes in mitral annulus area during the cardiac cycle, differently from other Carpentier types of MR, which were characterised by less dynamism. These findings were supported by previous CCTA studies on mitral valve prolapse [18] and functional MR [25, 32].

The low dynamic changes found in all Carpentier types except in type II might be more evident in our study compared to previous ones [25, 32], probably due to high prevalence of atrial fibrillation (30, 59%), which is likely to reduce the magnitude of dynamic changes of the mitral annulus during the cardiac cycle. Minimum annular dimension was found in the early systolic phase, caused by the active anteroposterior annulus contraction aimed to avoid early regurgitation [18, 20]. TT distance was not significantly enlarged in MR patients; this can be explained by the fact that the trigone zone and aorto-mitral curtain mainly consist of fibrotic tissue and thus being less prone to dilation, contrary to the posterior mitral annulus which is mainly muscular and consequently less resistant [20].

Mitral valve segmentation was performed using the D-shaped model, which is more accurate and reproducible than the saddle-shaped model in depicting the mitral annulus in MR patients [20, 24], and the planar landing zone of TMVI device [23, 31, 32]. In agreement with previous CCTA studies [20, 24], excellent accuracy of mitral annulus segmentation was found.

This study has some limitations: first, the relatively limited sample size. However, TMVI is a quite recent procedure for dedicated patients, therefore is performed in a limited and selected number of candidates. Only few patients with type I MR were included, reflecting the limited suitability of this category to these types of percutaneous treatment. Moreover, the control group consisted of patients without mitral valve disease who underwent multiphase CCTA for TAVR planning. Calcification of the aorto-mitral curtain determined a smaller mitral annulus area [44]; therefore, only patients with normal cardiac chamber volumes and function with no valvular calcification were included. Moreover, mitral annulus area and diameters in our control group were in line with previous data on healthy subjects [25], showing small valve area, delayed proto-diastolic peak, stable diastolic values, and a more efficient pre-systolic contraction with preserved contractility. A multiphase ECG-gated CCTA scan of the entire cardiac cycle cannot be justified in healthy subjects due to radiation exposure issue. Finally, our analysis is limited to the evaluation of only mitral valve annulus during the cardiac cycle. However, in the setting of TMVI, a number of additional parameters could be extracted from CCTA including calcification, leaflet tethering, abnormalities of papillary muscle insertion or anatomy, LVOT, regurgitant fraction, and percutaneous access [45].

In conclusion, our results showed largest annulus area mostly in systolic phases independently from the Carpentier MR type, with greater dynamic changes of the mitral annulus during the cardiac cycle in mitral valve prolapse compared to the other pathological conditions, which, on the contrary, were less dynamic.

Based on these findings, further research is required with aim of reducing radiation exposure. In fact, a multiphase ECG-gated CCTA acquisition with dose modulation should be considered. This would allow us to obtain full dose image acquisition in systole, necessary for better image definition and more accurate sizing, with reduced dose during the remaining phase of the cardiac cycle that could be however used to evaluate dynamic changes of cardiac structures, cardiac chamber volumes, and function.

## Data Availability

The datasets used and/or analysed during the current study are available from the corresponding author on reasonable request.

## Abbreviations

BSA: Body surface area
CCTA: Cardiac computed tomography angiography
CI: Confidence interval
ECG: Electrocardiogram
EF: Ejection fraction
IC: Intercommissural diameter
ICC: Intraclass correlation coefficient
LA: Left atrium
LA-ESV: Left atrial end-systolic volume
LV: Left ventricle
LV-EDV: Left ventricular end-diastolic volume
LV-EF: Left ventricular ejection fraction
LVOT: Left ventricle outflow tract
MR: Mitral valve regurgitation
SL: Septal-to-lateral diameter
TMVI: Transcatheter mitral valve implantation
TOE: Transoesophageal echocardiography
TT: Trigone-to-trigone distance
TTE: Transthoracic echocardiography

## References

[1] Iung B, Baron G, Butchart EG, Delahaye F, Gohlke-Bärwolf C, Levang OW, Tornos P, Vanoverschelde JL, Vermeer F, Boersma E, Ravaud P, Vahanian A (2003). A prospective survey of patients with valvular heart disease in Europe: the Euro Heart Survey on valvular heart disease. Eur Heart J.

[2] Vahanian A, Alfieri O, Andreotti F (2012). Guidelines on the management of valvular heart disease (version 2012). Eur Heart J.

[3] Markham R, Kyranis S, Aroney N, Lau K, Poon K, Scalia G, Walters D (2018). Transcatheter mitral valve intervention: an emerging treatment for mitral regurgitation. Intern Med J.

[4] Zoghbi WA, Adams D, Bonow RO, Enriquez-Sarano M, Foster E, Grayburn PA, Hahn RT, Han Y, Hung J, Lang RM, Little SH, Shah DJ, Shernan S, Thavendiranathan P, Thomas JD, Weissman NJ (2017). Recommendations for noninvasive evaluation of native valvular regurgitation: a report from the American Society of Echocardiography developed in collaboration with the Society for Cardiovascular Magnetic Resonance. J Am Soc Echocardiogr.

[5] Enriquez-Sarano M, Avierinos J-F, Messika-Zeitoun D, Detaint D, Capps M, Nkomo V, Scott C, Schaff HV, Tajik AJ (2005). Quantitative determinants of the outcome of asymptomatic mitral regurgitation. N Engl J Med.

[6] Mirabel M, Iung B, Baron G, Messika-Zeitoun D, Detaint D, Vanoverschelde JL, Butchart EG, Ravaud P, Vahanian A (2007). What are the characteristics of patients with severe, symptomatic, mitral regurgitation who are denied surgery?. Eur Heart J.

[7] Vahanian A, Urena M, Ince H, Nickenig G (2017). Mitral valve: repair/clips/cinching/chordae. EuroIntervention.

[8] De Bonis M, Al-Attar N, Antunes M (2016). Surgical and interventional management of mitral valve regurgitation: a position statement from the European Society of Cardiology working groups on cardiovascular surgery and valvular heart disease. Eur Heart J.

[9] Wang DD, Eng MH, Greenbaum AB, Myers E, Forbes M, Karabon P, Pantelic M, Song T, Nadig J, Guerrero M, O'Neill WW (2018). Validating a prediction modeling tool for left ventricular outflow tract (LVOT) obstruction after transcatheter mitral valve replacement (TMVR). Catheter Cardiovasc Interv.

[10] Lee JZ, Tey KR, Mizyed A, Hennemeyer CT, Janardhanan R, Lotun K (2015). Mitral valve replacement complicated by iatrogenic left ventricular outflow obstruction and paravalvular leak: case report and review of literature. BMC Cardiovasc Disord.

[11] Gheorghe LL, Mobasseri S, Agricola E, Wang DD, Milla F, Swaans M, Pandis D, Adams DH, Yadav P, Sievert H, Ailawadi G, Sorajja P (2021). Imaging for native mitral valve surgical and transcatheter interventions. JACC Cardiovasc Imaging.

[12] Ranganath P, Moore A, Guerrero M, Collins J, Foley T, Williamson E, Rajiah P (2020). CT for pre-and postprocedural evaluation of transcatheter mitral valve replacement. Radiographics.

[13] Guerrero M, Wang DD, Pursnani A, Eleid M, Khalique O, Urena M, Salinger M, Kodali S, Kaptzan T, Lewis B, Kato N, Cajigas HM, Wendler O, Holzhey D, Pershad A, Witzke C, Alnasser S, Tang GHL, Grubb K, Reisman M, Blanke P, Leipsic J, Williamson E, Pellikka PA, Pislaru S, Crestanello J, Himbert D, Vahanian A, Webb J, Hahn RT, Leon M, George I, Bapat V, O’Neill W, Rihal C (2020). A cardiac computed tomography–based score to categorize mitral annular calcification severity and predict valve embolization. JACC Cardiovasc Imaging.

[14] Faggioni L, Gabelloni M, Accogli S, Angelillis M, Costa G, Spontoni P, Petronio AS, Caramella D (2018). Preprocedural planning of transcatheter mitral valve interventions by multidetector CT: what the radiologist needs to know. Eur J Radiol Open.

[15] Blanke P, Naoum C, Dvir D, Bapat V, Ong K, Muller D, Cheung A, Ye J, Min JK, Piazza N, Theriault-Lauzier P, Webb J, Leipsic J (2017). Predicting LVOT obstruction in transcatheter mitral valve implantation: concept of the Neo-LVOT. JACC Cardiovasc Imaging.

[16] Blanke P, Naoum C, Webb J, Dvir D, Hahn RT, Grayburn P, Moss RR, Reisman M, Piazza N, Leipsic J (2015). Multimodality imaging in the context of transcatheter mitral valve replacement establishing consensus among modalities and disciplines. JACC Cardiovasc Imaging.

[17] Grover R, Ohana M, Arepalli CD, Sellers SL, Mooney J, Kueh SH, Kim U, Blanke P, Leipsic JA (2018). Role of MDCT imaging in planning mitral valve intervention. Curr Cardiol Rep.

[18] Van Wijngaarden SE, Kamperidis V, Regeer MV (2018). Three-dimensional assessment of mitral valve annulus dynamics and impact on quantification of mitral regurgitation. Eur Heart J Cardiovasc Imaging.

[19] Pouch AM, Vergnat M, McGarvey JR (2014). Statistical assessment of normal mitral annular geometry using automated three-dimensional echocardiographic analysis. Ann Thorac Surg.

[20] Noack T, Janietz M, Lurz P, Kiefer P, Sieg F, Marin-Cuartas M, Spampinato R, Besler C, Rommel KP, Holzhey D, Mohr FW, Ender J, Borger MA, Seeburger J (2019). Dynamic mitral valve geometry in patients with primary and secondary mitral regurgitation: implications for mitral valve repair†. Eur J Cardio-Thoracic Surg.

[21] Aruta P, Muraru D, Guta AC, Mihaila S, Ruozi N, Palermo C, Elnagar B, Iliceto S, Badano LP (2018). Comparison of mitral annulus geometry between patients with ischemic and non-ischemic functional mitral regurgitation: implications for transcatheter mitral valve implantation. Cardiovasc Ultrasound.

[22] Faletra FF, Leo LA, Paiocchi VL, Caretta A, Viani GM, Schlossbauer SA, Demertzis S, Ho SY (2019). Anatomy of mitral annulus insights from non-invasive imaging techniques. Eur Heart J Cardiovasc Imaging.

[23] Naoum C, Leipsic J, Cheung A, Ye J, Bilbey N, Mak G, Berger A, Dvir D, Arepalli C, Grewal J, Muller D, Murphy D, Hague C, Piazza N, Webb J, Blanke P (2016). Mitral annular dimensions and geometry in patients with functional mitral regurgitation and mitral valve prolapse implications for transcatheter mitral valve implantation. JACC Cardiovasc Imaging.

[24] Alkadhi H, Desbiolles L, Stolzmann P, Leschka S, Scheffel H, Plass A, Schertler T, Trindade PT, Genoni M, Cattin P, Marincek B, Frauenfelder T (2009). Mitral annular shape, size, and motion in normals and in patients with cardiomyopathy: evaluation with computed tomography. Invest Radiol.

[25] Rizvi A, Marcus RP, Guo Y, Carter R, Mark IT, Foley TA, Weber NM, Sheedy EN, Leng S, Williamson EE (2020). Dynamic computed tomographic assessment of the mitral annulus in patients with and without mitral prolapse. J Cardiovasc Comput Tomogr.

[26] Banks T, Razeghi O, Ntalas I, Aziz W, Behar JM, Preston R, Campbell B, Redwood S, Prendergast B, Niederer S, Rajani R (2018). Automated quantification of mitral valve geometry on multi-slice computed tomography in patients with dilated cardiomyopathy – implications for transcatheter mitral valve replacement. J Cardiovasc Comput Tomogr.

[27] Thériault-Lauzier P, Dorfmeister M, Mylotte D, Spaziano M, Blanke P, Martucci G, Lange R, Leipsic J, Bilodeau L, Piazza N, Andalib A (2016). Quantitative multi-slice computed tomography assessment of the mitral valvular complex for transcatheter mitral valve interventions part 2: geometrical measurements in patients with functional mitral regurgitation. EuroIntervention.

[28] Koo HJ, Yang DH, Oh SY, Kang JW, Kim DH, Song JK, Lee JW, Chung CH, Lim TH (2014). Demonstration of mitral valve prolapse with CT for planning of mitral valve repair. Radiographics.

[29] Baumgartner H, Falk V, Bax JJ, de Bonis M, Hamm C, Holm PJ, Iung B, Lancellotti P, Lansac E, Rodriguez Muñoz D, Rosenhek R, Sjögren J, Tornos Mas P, Vahanian A, Walther T, Wendler O, Windecker S, Zamorano JL, Roffi M, Alfieri O, Agewall S, Ahlsson A, Barbato E, Bueno H, Collet JP, Coman IM, Czerny M, Delgado V, Fitzsimons D, Folliguet T, Gaemperli O, Habib G, Harringer W, Haude M, Hindricks G, Katus HA, Knuuti J, Kolh P, Leclercq C, McDonagh TA, Piepoli MF, Pierard LA, Ponikowski P, Rosano GMC, Ruschitzka F, Shlyakhto E, Simpson IA, Sousa-Uva M, Stepinska J, Tarantini G, Tchétché D, Aboyans V, Windecker S, Aboyans V, Agewall S, Barbato E, Bueno H, Coca A, Collet JP, Coman IM, Dean V, Delgado V, Fitzsimons D, Gaemperli O, Hindricks G, Iung B, Jüni P, Katus HA, Knuuti J, Lancellotti P, Leclercq C, McDonagh T, Piepoli MF, Ponikowski P, Richter DJ, Roffi M, Shlyakhto E, Simpson IA, Zamorano JL, Kzhdryan HK, Mascherbauer J, Samadov F, Shumavets V, Camp GV, Lončar D, Lovric D, Georgiou GM, Linhartova K, Ihlemann N, Abdelhamid M, Pern T, Turpeinen A, Srbinovska-Kostovska E, Cohen A, Bakhutashvili Z, Ince H, Vavuranakis M, Temesvári A, Gudnason T, Mylotte D, Kuperstein R, Indolfi C, Pya Y, Bajraktari G, Kerimkulova A, Rudzitis A, Mizariene V, Lebrun F, Demarco DC, Oukerraj L, Bouma BJ, Steigen TK, Komar M, de Moura Branco LM, Popescu BA, Uspenskiy V, Foscoli M, Jovovic L, Simkova I, Bunc M, de Prada JAV, Stagmo M, Kaufmann BA, Mahdhaoui A, Bozkurt E, Nesukay E, Brecker SJD, ESC Scientific Document Group (2017). 2017 ESC/EACTS guidelines for the management of valvular heart disease. Eur Heart J.

[30] Pulerwitz TC, Khalique OK, Leb J, Hahn RT, Nazif TM, Leon MB, George I, Vahl TP, D’Souza B, Bapat VN, Dumeer S, Kodali SK, Einstein AJ (2020). Optimizing cardiac CT protocols for comprehensive acquisition prior to percutaneous MV and TV repair/replacement. JACC Cardiovasc Imaging.

[31] Blanke P, Dvir D, Cheung A, Ye J, Levine RA, Precious B, Berger A, Stub D, Hague C, Murphy D, Thompson C, Munt B, Moss R, Boone R, Wood D, Pache G, Webb J, Leipsic J (2014). A simplified D-shaped model of the mitral annulus to facilitate CT-based sizing before transcatheter mitral valve implantation. J Cardiovasc Comput Tomogr.

[32] Blanke P, Dvir D, Naoum C, Cheung A, Ye J, Thériault-Lauzier P, Spaziano M, Boone RH, Wood DA, Piazza N, Webb JG, Leipsic J (2015). Prediction of fluoroscopic angulation and coronary sinus location by CT in the context of transcatheter mitral valve implantation. J Cardiovasc Comput Tomogr.

[33] Lang RM, Badano LP, Victor MA (2015). Recommendations for cardiac chamber quantification by echocardiography in adults: an update from the American Society of Echocardiography and the European Association of Cardiovascular Imaging. J Am Soc Echocardiogr.

[34] Carpentier A, Adams D, Filsoufi F (2010). Carpentier’s Reconstructive Valve Surgery - 1st Edition.

[35] de Groot-de Laat LE, McGhie J, Ren B, Frowijn R, Oei FB, Geleijnse ML (2019). A modified echocardiographic classification of mitral valve regurgitation mechanism: the role of three-dimensional echocardiography. J Cardiovasc Imaging.

[36] El Sabbagh A, Reddy YNV, Nishimura RA (2018). Mitral valve regurgitation in the contemporary era: insights into diagnosis, management, and future directions. JACC Cardiovasc Imaging.

[37] Cicchetti DV (1994). Guidelines, criteria, and rules of thumb for evaluating normed and standardized assessment instruments in psychology. Psychol Assess.

[38] Nishimura RA, Vahanian A, Eleid MF, Mack MJ (2016). Mitral valve disease - current management and future challenges. Lancet.

[39] Bryhn M, Gåarding L (1986). The mitral valve mechanism with normal and prolapsed leaflets in the light of a dynamic model. Clin Cardiol.

[40] Lee APW, Jin CN, Fan Y, Wong RHL, Underwood MJ, Wan S (2017). Functional implication of mitral annular disjunction in mitral valve prolapse: a quantitative dynamic 3D echocardiographic study. JACC Cardiovasc Imaging.

[41] Sonne C, Sugeng L, Watanabe N, Weinert L, Saito K, Tsukiji M, Yoshida K, Takeuchi M, Mor-Avi V, Lang RM (2009). Age and body surface area dependency of mitral valve and papillary apparatus parameters: assessment by real-time three-dimensional echocardiography. Eur J Echocardiogr.

[42] Mihǎilǎ S, Muraru D, Piasentini E (2014). Quantitative analysis of mitral annular geometry and function in healthy volunteers using transthoracic three-dimensional echocardiography. J Am Soc Echocardiogr.

[43] Enriquez-Sarano M (2017). Mitral annular disjunction: the forgotten component of myxomatous mitral valve disease. JACC Cardiovasc Imaging.

[44] Tsang W, Meineri M, Hahn RT, Veronesi F, Shah AP, Osten M, Nathan S, Russo M, Lang RM, Horlick EM (2013). A three-dimensional echocardiographic study on aortic-mitral coupling in transcatheter aortic valve replacement. Eur Heart J Cardiovasc Imaging.

[45] Weir-McCall JR, Blanke P, Naoum C (2018). Mitral valve imaging with CT: relationship with transcatheter mitral valve interventions. Radiology.

